# Effects of Pre-Cooking Degree of Germinated Highland Barley Pulp on the Quality and Digestive Characteristics of Barbecued Pork Buns During Refrigerated Storage

**DOI:** 10.3390/foods15101775

**Published:** 2026-05-18

**Authors:** Yuying Cheng, Zheng Ruan, Jian Yang, Zhexi Weng, Biansheng Li, Dandan Li, Jiaqin Fang

**Affiliations:** 1 Guangzhou Restaurant Group Likoufu Food Co., Ltd., Guangzhou 511445, China; chengyy430@163.com (Y.C.); lkfkjxm@126.com (J.Y.); wengzzxx@163.com (Z.W.); 2 School of Food Science and Engineering, South China University of Technology, Guangzhou 510640, China; febshli@scut.edu.cn (B.L.); feddli@scut.edu.cn (D.L.); 3 Guangdong Provincial Key Laboratory of Green Processing of Natural Products and Product Safety, Guangzhou 510640, China

**Keywords:** germinated highland barley pulp, barbecued pork buns, refrigerated storage, staling properties, digestive properties

## Abstract

This study investigated the effect of pre-cooking level of germinated highland barley pulp on the staling properties and digestibility of pre-packaged barbecued pork buns during refrigerated storage (0–9 days). The addition of barley pulp significantly delayed quality deterioration, resulting in a decreased specific volume (up to 20%) and an increased hardness (up to 71.76%) across all samples. Furthermore, it effectively inhibited the rise in starch short-range order, as evidenced by a decreased FTIR ratio of 1047/1022 cm^−1^, and retarded the conformational transition between protein α-helix and β-sheet structures. When the gelatinization degree increased to 91.22%, rapidly digestible starch (RDS) decreased significantly while resistant starch (RS) increased. The sauce infiltration layer exhibited a higher maximum RS (23.23%) than the inner crumb (16.52%). The Glycemic Index (GI) was significantly reduced, with the lowest values observed in the BJ60 group (53.22 for the sauce infiltration layer and 60.37 for the inner crumb). α-Amylase inhibition was also enhanced with increasing gelatinization degrees. Significant correlations were found between starch structural parameters and digestibility. These results demonstrate that incorporating germinated highland barley pulp is a feasible strategy to simultaneously improve the shelf-life and nutritional quality of steamed buns.

## 1. Introduction

Highland barley, a unique cereal resource native to China’s Qinghai-Tibet Plateau region, is characterized by its high dietary fiber (12–15% total dietary fiber, dry basis), high vitamin content, and elevated levels of β-glucans. Its starch comprises 74–78% amylopectin, reaching up to 100% in certain varieties. Germination activates endogenous enzymes (α-amylase, protease, β-glucanase), which partially degrade starch and proteins, increase γ-aminobutyric acid (GABA) content (from 28 to 86 mg/100 g), and reduce anti-nutritional factors such as phytic acid (by ~23%) [1]. Owing to these properties, germinated highland barley demonstrates beneficial effects in maintaining intestinal health, regulating blood lipids and glucose, and preventing cardiovascular and cerebrovascular diseases [2].

During the processing of highland barley, pre-cooking is a critical step for enhancing its edible quality and functional properties. This treatment induces starch gelatinization, reduces moisture content, improves digestibility, and simultaneously enhances storage stability and safety [3]. Steam cooking, as a typical moist-heat processing method, is conducted at medium temperatures of 100–200 °C. It facilitates starch gelatinization through water penetration and induces moderate protein denaturation, offering advantages such as simple operation and low energy consumption. Research indicates that steam cooking not only improves the process adaptability of highland barley but also significantly influences its nutritional and health benefits. Li Yongfu et al. [4] found through animal experiments that steam-cooked highland barley products significantly reduced serum total cholesterol levels in hyperlipidemic model rats, with a reduction of 18–25%. In a study on highland barley brewing, Kok et al. [5] applied steam cooking at 121 °C for 30 min, achieving a starch gelatinization degree of over 92%, effectively inhibiting the formation of retrograde starch. This approach simplified the traditional saccharification process, shortened the fermentation time by 24–36 h, and increased alcohol yield by 5–8%. On the other hand, steam cooking may also induce changes in certain components of highland barley. Li Limei et al. [6] reported that this treatment led to a 15–20% decrease in water-soluble protein content and an 8–12% increase in lipid oxidation. In vitro digestion experiments further revealed that the digestibility rate constant of highland barley starch decreased by 25–30% after steam cooking. Pre-cooking (steaming) gelatinizes starch, improves water-holding capacity, and enhances the functional properties (e.g., enzyme inhibition, gel formation). The combination of germination and pre-cooking maximizes the nutritional (higher GABA, lower phytate) and anti-staling (better water distribution, retrogradation inhibition) benefits in steamed bun products.

Barbecued pork buns, a representative Cantonese dim sum, faces core challenges in industrial production related to quality deterioration. During refrigeration, starch molecules undergo retrogradation, leading to a gradual increase in crust hardness over time [7]. Additionally, under refrigerated conditions, moisture continuously migrates to the crust, resulting in filling shrinkage and a softening-wet crust. After 7 days of refrigeration, the filling moisture loss rate reaches 12%, while the crust moisture content increases by 9%, leading to a 30% decline in the overall texture score of the product.

Although pre-cooking of highland barley has been studied for its effects on starch properties, and germination is known to enhance nutritional value, the combined effect of germination and pre-cooking degree on both staling properties (during refrigerated storage) and digestive characteristics (including GI and α-amylase inhibition) in a complex meat-filled steamed bun system has not been reported. This study aims to investigate the effects of the pre-cooking degree of germinated highland barley pulp on the staling and digestive properties of barbecued pork buns. Through refrigeration experiments, quality changes, alterations in starch and protein structures, as well as the starch hydrolysis rate, glycemic index (GI), and α-amylase inhibition of barbecued pork buns will be determined. The research seeks to elucidate the underlying mechanisms affecting the staling and digestibility characteristics, with the goal of developing highland barley barbecued pork buns that combines desirable eating quality with favorable digestive properties. Therefore, the objective of this study was to systematically investigate the effect of germinated highland barley pulp with varying pre-cooking degrees on the physicochemical staling properties, in vitro digestibility, and underlying structural changes of pre-packaged barbecued pork buns during refrigerated storage.

## 2. Materials and Methods

### 2.1. Main Materials and Reagents

Wheat flour (wet gluten content 26.60%, moisture content 12.80%) and highland barley were provided by Guangzhou Restaurant Group Likofu Food Co., Ltd. (Guangzhou, China). High-active dry yeast was obtained from Angel Yeast Co., Ltd. (Yichang, China). Edible salt was provided by Fujian Province Zhongyan Group Co., Ltd. (Xiamen, China). Potassium bromide (KBr), absolute ethanol, anhydrous glucose, anhydrous sodium acetate, and glacial acetic acid were all of analytical grade. DNS color developing solution was acquired from Feijing Biotechnology Co., Ltd. (Fuzhou, China). Porcine pancreatic α-amylase and amylo-glucosidase were sourced from Shanghai Yuanye Bio-Technology Co., Ltd. (Shanghai, China).

### 2.2. Experimental Methods

#### 2.2.1. Preparation of Germinated Highland Barley Pulp

Germination of Highland barley: highland barley grains were sorted to remove moldy, immature, broken grains, and impurities, followed by washing and transfer into a germination tank. They were then soaked in sterile water for 3–4 h, after which the sterile water was drained. After draining, the grains were germinated at 25–30 °C and 95–99% relative humidity for 24 h in the dark, targeting a radicle length of 0.5–1.0 mm. Throughout the process, rotational micro-aeration was maintained via low-speed tumbling every 2–3 h and atomized misting every 1–3 h to optimize moisture and oxygen distribution [8].

Pre-cooking treatment: the germinated highland barley was steamed using an HC-400 steam cabinet (Guangzhou Hengxin Western Kitchen Equipment Co., Ltd., Guangzhou, China) at a steam flow rate of 15.0 mL/min for durations of 10 min, 30 min, 60 min, and 90 min, respectively. Timing commenced once the water temperature reached 100 °C. The heat treatment load applied was 1.50–2.00 kJ·kg^−1^·s^−1^ (wet basis). Subsequently, the steamed grains were coarsely ground using a wall breaker and then finely ground with a W30 electric stone mill (Foshan Weimeishi Electric Appliance Technology Co., Ltd., Foshan, China). Germinated highland barley pulp with varying degrees of gelatinization was prepared at a solid-to-liquid ratio of 1:3, and the samples were designated as BJ10, BJ30, BJ60, and BJ90, with gelatinization degrees of 33.37%, 58.25%, 87.90%, and 91.22%, respectively. The untreated (unsteamed) germinated highland barley pulp was labeled as AJ0.

#### 2.2.2. Preparation of Barbecued Pork Bun Samples

The wrapper formulation was formulated with 500 g of wheat flour as the base. According to the baking percentage, the mass fraction of added barley pulp solids was 8%. The mass fraction of added water was determined based on farinograph characteristics. Other ingredients included 5 g of dry yeast, 80 g of white sugar, 5.5 g of leavening agent, and 1 g of salt. The barbecued pork with sauce was used as the filling. The skin-to-filling ratio was maintained at 2:1. Inner crumb is the central part of the bun (excluding crust and filling). Sauce infiltration layer is the 3-mm thick layer of bun crumb immediately adjacent to the filling that has absorbed sauce. Each bun (Sample) was separated into two fractions: the inner crumb (designated as −1) and the sauce infiltration layer (designated as −2).

Mixing was performed using a vertical mixer: initially at a slow speed for 4 min (131 rpm), followed by fast mixing for 6 min (262 rpm). The dough was then sheeted using an automatic dough sheeter for 100 s. Proofing was carried out in a proofing box for 50 min at the temperature of 37–39 °C and the relative humidity of 70–75%. Steaming was conducted under full load in a HC-400 steam cabinet at 0.025 MPa/100 °C for 600 s.

Barbecued pork buns prepared without barley pulp were designated as the “control”. All samples were stored at 4 °C in a refrigerator (KG32 NV21 EC; BSH Home Appliances Co., Ltd., Chuzhou, Anhui, China) for 0, 3, 5, 7, and 9 days, respectively.

#### 2.2.3. Determination of Quality Changes During Refrigeration

Specific Volume: The specific volume was determined using the rapeseed displacement method in accordance with Chinese National Standard GB/T 21118-2007 [9]. The mass of each bun was measured with a BSA224 S analytical balance (Sartorius AG, Göttingen, Germany; sensitivity: 0.01 g). The specific volume (mL/g) was calculated as the volume divided by the mass.

Hardness: The whole bun was placed on the platform of a TA-XT Plus Texture Analyzer (Stable Micro Systems Ltd., Godalming, UK) [10]. The test settings were configured as follows: a P/100 probe was used, with a pre-test speed of 5.0 mm/s, test speed of 1.0 mm/s, and post-test speed of 5.0 mm/s. A compression strain of 50% was applied, with a trigger force of 5 g and a time interval of 5 s between two compressions. Textural properties including hardness, springiness, chewiness, and cohesiveness were measured.

#### 2.2.4. Mechanism Analysis of Refrigeration Quality Change

Starch Relative Crystallinity: Inner crumb samples from buns subjected to different refrigeration periods were freeze-dried (DHG-9070A; Shanghai Shenxian Thermostatic Equipment Factory, Shanghai, China) and ground into powder [11]. Crystalline structure was analyzed with an X’pert Powder X-ray diffractometer (Panalytical B.V., Almelo, The Netherlands; Cu-Kα radiation) operated at 40 kV and 40 mA. Scans were performed from 4° to 40° (2θ) at a rate of 4°/min with a step size of 0.02°. Relative crystallinity (%) was calculated from the XRD patterns using MDI Jade 6.5 software (Materials Data, Inc., Livermore, CA, USA).

Starch Short-Range Order: Freeze-dried powder from the inner crumb was blended with pre-dried KBr (105 °C, 48 h) at a 1:60 (*w*/*w*) ratio. After drying and grinding, pellets were prepared and analyzed with a Nicoletis-50 FTIR spectrometer (Thermo Fisher Scientific (China) Co., Ltd., Shanghai, China) [12]. Spectra were acquired from 400 cm^−1^ to 4000 cm^−1^ at 4 cm^−1^ resolution with 32 scans. The region 1200–800 cm^−1^ was processed by second-derivative deconvolution using OMNIC 8.2 software to improve band resolution. The intensity ratio of 1047 cm^−1^ to 1022 cm^−1^ was determined.

Protein Secondary Structure: Protein secondary structure was analyzed using the same Nicoletis-50 FTIR spectrometer, with sample treatment and spectral range consistent with Starch Short-Range Order [13]. The amide I band (1600–1700 cm^−1^) was processed via baseline correction, Fourier self-deconvolution, second-derivative analysis, and Gaussian curve fitting using PeakFit 4.12 software to quantify structural components.

#### 2.2.5. Determination of Nutritional Characteristics

In vitro Digestibility: The in vitro digestibility was determined according to the method described by Warren et al. [14] with modifications. Anhydrous glucose was dried at 105 °C for 2 h and used to prepare a series of standard solutions ranging from 0.2 to 1.0 mg/mL. Then, 200 μL of each solution was mixed with 400 μL of DNS reagent, heated in a boiling water bath for 5 min, and cooled in an ice bath. The mixture was diluted to 5 mL with distilled water, and the absorbance was measured at 540 nm using distilled water as a blank. A standard curve was plotted based on the measured values.

The inner crumb of barbecued pork bun or the sauce infiltration layer (1 g, 3 mm thick) was weighed and mixed with 20 mL of 0.2 mol/L sodium acetate buffer (pH 5.2). The mixture was pre-incubated in a 37 °C water bath for 10 min. Then, 5 mL of an enzyme cocktail containing 1450 U of porcine pancreatic α-amylase and 75 U of amyloglucosidase was added, and enzymatic hydrolysis was performed at 37 °C with shaking at 150 r/min. At 0, 20, 40, 60, 90, 120, and 180 min, 1 mL aliquots of the digest were transferred into centrifuge tubes containing 4 mL of absolute ethanol to terminate the reaction. The mixtures were centrifuged at 8000 r/min for 10 min, and the supernatant was collected for glucose content determination using the DNS colorimetric method. The contents of RDS, SDS, and RS were calculated based on the glucose released at different time points during in vitro digestion (20, 120, and 180 min) following the Englyst method. Total starch was analyzed using AOAC Method 996.11 (Total Starch Assay Kit; Megazyme Ltd., Bray, Ireland) prior to the digestibility measurement.

Glycemic Index (GI) Estimation: An in vitro starch digestion kinetic model was established for the analysis of either the inner crumb or the sauce infiltration layer (3 mm thick) of the barbecued pork buns, based on the nonlinear equation proposed by Goñi et al. [15]. In this model, the starch hydrolysis percentage at a given time (C) is mathematically related to the equilibrium hydrolysis percentage reached at 180 min (C∞) and a kinetic constant (k). The parameters C∞ and k for different samples were determined by fitting the model to the data obtained from the in vitro digestion experiments.

The Hydrolysis Index (HI), a significant indicator for predicting the glycemic response of food, was calculated as the ratio of the area under the hydrolysis curve (AUC) for the experimental group to the AUC of the inner crumb from the control bun. The Glycemic Index (GI) was then estimated using the calculation method introduced by Ferrer et al. [16].

α-Amylase Inhibitory Activity Assay: The α-amylase inhibitory activity was determined according to the method of Wu et al. [17] with modifications. Briefly, 40 mL of soluble starch solution (0.5 mg/mL) was added to a conical flask, preheated at 37 °C for 5 min. Then, 1 g of the inner crumb or sauce infiltration layer (3 mm thick) of the barbecued pork bun and 3 mL of α-amylase solution (500 U/mL) were added, and the reaction was carried out with shaking for 30 min. Subsequently, 4 mL of the digestion suspension was immediately inactivated in a boiling water bath, followed by centrifugation at 6000 r/min for 10 min. The reducing sugar content in the supernatant was determined using the DNS method. A positive control (3 mL α-amylase + 40 mL starch solution) and a negative control (1 g sample + 40 mL starch solution) were established. The α-amylase inhibition rate was calculated by comparing the reducing sugar content of the sample group with those of the positive and negative controls, reflecting the inhibitory capacity of the sample on enzyme activity.

#### 2.2.6. Data Analysis

All data were processed using Excel 2019, SPSS 22.0, and Origin 2022 software. Significant differences were analyzed by Duncan’s new multiple range test at a 95% confidence level (*p* < 0.05). The results are expressed as “mean ± standard deviation”.

## 3. Results and Discussion

### 3.1. Quality Changes of Germinated Highland Barley Pulp Buns During Refrigeration

**Figure 1:**
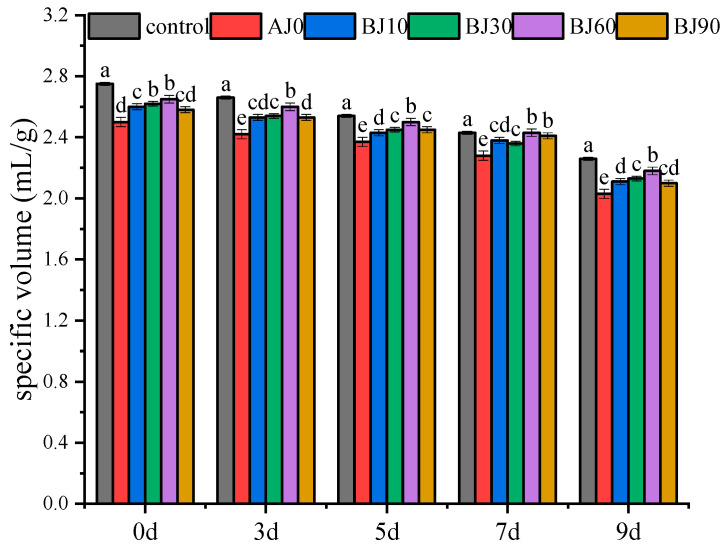
The effect of germinated highland barley pulp on the specific volume of barbecued pork bun during storage, different letters indicate significant differences (*p* < 0.05).

The staling of barbecued pork buns during refrigerated storage significantly reduces their specific volume, a key parameter directly related to product quality. As shown in Figure 1, the addition of germinated highland barley pulp resulted in a lower specific volume compared to the control group, likely due to disruption of the gluten network, leading to reduced gas retention and fermentation capacity. As the gelatinization degree of the barley pulp increased, the specific volume initially increased and then decreased, with the BJ60 group (medium gelatinization degree) exhibiting the highest specific volume. This suggests that pre-cooking can soften the insoluble dietary fiber in highland barley, enabling it to form a dense network that encapsulates starch granules and improves the loose gluten structure. During refrigeration, the specific volume of all samples decreased significantly (*p* < 0.05): the control group decreased from 2.75 mL/g to 2.20 mL/g (20% reduction), while the reduction in other experimental groups ranged from 18.29% and 18.56%. This decrease was primarily attributed to moisture migration and starch retrogradation. The resulting rearrangement of starch molecules increases gel hardness and reduces elasticity, disrupting the pore structure and allowing gas to escape. Similar observations linking starch retrogradation, moisture migration, and pore structure disruption in frozen flour products have been reported by Liu [12]. Notably, although the addition of barley pulp generally reduced the specific volume, it slowed the rate of decrease during refrigeration, indicating that barley pulp can inhibit staling and retrogradation. Furthermore, the degree of gelatinization had a graded effect on the extent of staling. For instance, the optimized BJ60 treatment likely formed a more stable supporting framework within the dough structure by modulating the interactions between dietary fiber and starch.

**Figure 2:**
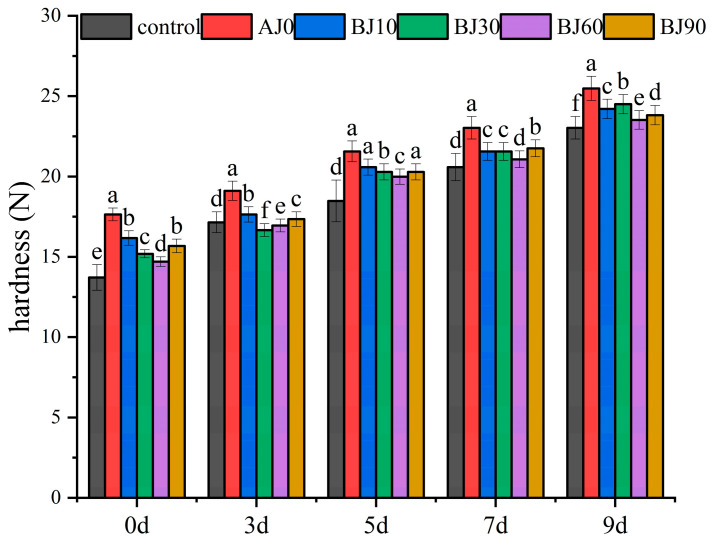
The effect of germinated highland barley pulp on the hardness of barbecued pork bun during storage, different letters indicate significant differences (*p* < 0.05).

Staling readily occurs in barbecued pork buns during refrigeration, primarily caused by complex changes such as amylopectin retrogradation and moisture loss, which manifest as texture hardening, taste deterioration, and reduced acceptability [18]. As shown in Figure 2, texture hardness is a core indicator of staling: the hardness of buns with added barley pulp was higher than that of the control group, due to the strong water-holding capacity of the dietary fiber, which absorbs free water and alters water distribution. The hardness of all samples increased significantly with refrigeration time (*p* < 0.05), and the increase rate from day 5 to 9 was higher than that from day 0 to 5. The hardness of the control group increased by 71.76%, while the increases for the different gelatinization degree groups ranged from 47.56% to 59.36%. During starch staling, amylose molecules form double helices via hydrogen bonding, these helices then aggregate into crystalline regions, leading to increased crystallinity and accelerated hardening [19]. However, the addition of barley pulp reduced the rate of hardness increase, indicating its ability to inhibit staling by regulating the rearrangement of starch molecules. Moreover, the retarding effect on staling varied with the degree of gelatinization (e.g., the high gelatinization group exhibited the lowest rate of increase). These results are consistent with the thermomechanical property analysis of the dough, confirming that barley pulp forms a stable structure through the interaction between dietary fiber and starch. This structure helps maintain water balance while interfering with starch crystallization, thereby delaying staling and retrogradation.

### 3.2. Changes in Starch Relative Crystallinity

**Figure 3:**
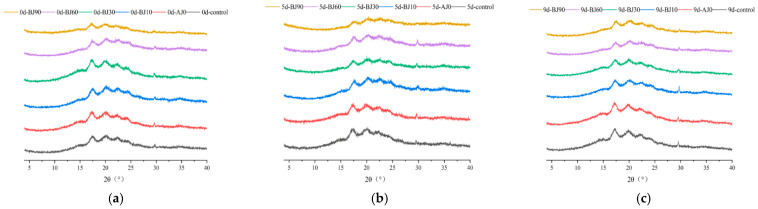
X-ray diffraction patterns of the inner crumb of germinated highland barley pulp barbecued pork bun during storage. (**a**) refers to refrigeration of 0 d; (**b**) 5 d; (**c**) 9 d.

Starch crystallinity is primarily determined by the integrity of the amylopectin double-helix structure and intermolecular hydrogen bonding, serving as a key parameter for characterizing starch crystal properties [20]. As shown in Figure 3, X-ray diffraction analysis of the long-range ordered structure of starch revealed distinct diffraction peaks at 2θ = 15°, 17°, and 23°, characteristic of the typical A-type crystalline structure of cereals, and a peak at 2θ = 20°, indicating a V-type structure composed of single helices, resulting in an overall A + V-type crystal characteristic [21]. After the adding of barley pulp, the diffraction peak intensity decreased with increasing gelatinization degree. At day 0, the relative crystallinity of the control group was 19.47%, while that of the barley pulp groups with increasing gelatinization degree (AJ0 to BJ90) decreased to 18.66%, 17.73%, 16.11%, 15.58%, and 13.50%, respectively, representing reductions of 0.81% to 5.97%. It is important to note that the X-ray diffraction signal originates primarily from wheat starch in the bun, as barley pulp solids account for only 8% of the total flour solids. Therefore, the observed decrease in relative crystallinity reflects reduced ordering of the wheat starch rather than a direct measurement of barley starch crystallinity. This effect is attributed to the physical interference of barley dietary fiber and pregelatinized barley components with wheat starch gelatinization during steaming. Specifically, the hydrothermal conditions (100 °C, 600 s) cause wheat starch granules to swell, but the presence of barley fiber and pregelatinized starch restricts the reorganization of starch chains. This disruption of the hydrogen bond network limits chain reassociation upon cooling, resulting in a more disordered amorphous state, as reflected by the lower relative crystallinity.

During refrigeration, the relative crystallinity of all samples increased significantly (*p* < 0.05). By day 9, the relative crystallinity of the control group had increased by 5.23%, while increases in the barley pulp treatment groups ranged from 3.75% to 5.04% (highest in BJ30, lowest in BJ90). This increase is attributed to the formation of double helices through hydrogen bonding between amylose molecules, which aggregate into crystalline regions and accelerate the staling process. However, the addition of barley pulp significantly suppressed the increase in crystallinity. For example, the BJ90 group exhibited only a 3.83% increase in relative crystallinity after 9 days of refrigeration, indicating that barley pulp delays staling by interfering with the ordered arrangement of starch molecules. Mechanistically, the network of gelatinized starch and dietary fiber in the barley pulp may encapsulate starch granules, thereby inhibiting moisture migration and molecular chain rearrangement. Furthermore, a higher degree of gelatinization causes greater disruption of the original crystal structure (e.g., BJ90 had the lowest initial crystallinity). These results confirm that barley pulp can gradually slow the staling rate of barbecued pork buns by regulating starch crystallization kinetics.

### 3.3. Changes in Starch Short-Range Order

**Figure 4:**
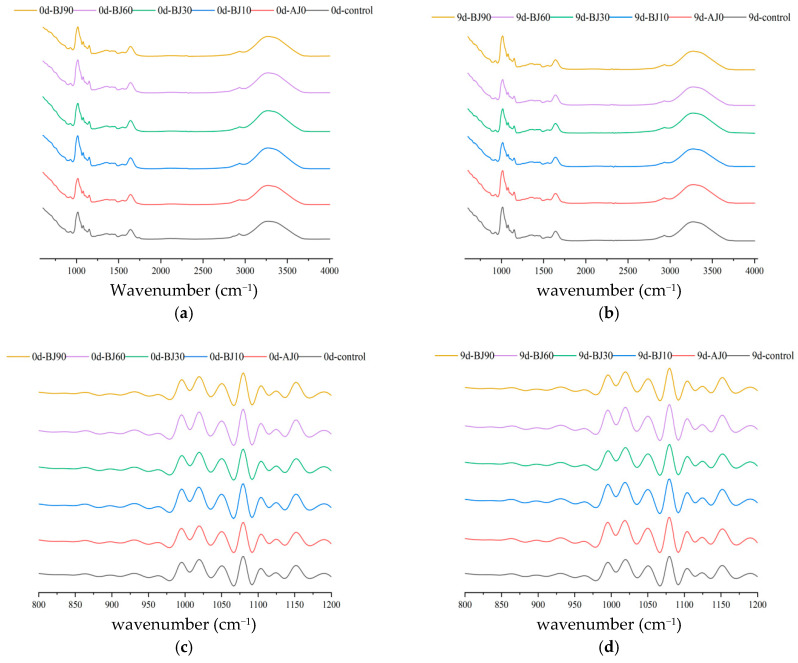
Infrared spectra of starch in the inner crumb of the germinated highland barley pulp barbecued pork bun during storage (**a**,**b**) and deconvolution graphs (**c**,**d**), (**a**,**c**) were refrigerated of 0 d; (**b**,**d**) 9 d.

Fourier Transform Infrared Spectroscopy (FTIR) can analyze the short-range ordered structure of starch, revealing changes in chain conformation and helical structure during staling (Figure 4). The broad absorption band around 3200–3500 cm^−1^ corresponds to O-H stretching vibration, the multiple peaks in the 3000–2800 cm^−1^ region are attributed to -CH_2_/CH_3_ group vibrations, the peak at 1640–1643 cm^−1^ is due to O-H bending vibration of bound water, and the peaks around 1016 cm^−1^ and 1151 cm^−1^ belong to C-O stretching vibrations. The spectra of the barley pulp groups were similar to that of the control, with no significant shift in characteristic peak positions during staling [22].

Deconvolution analysis reveals that the ratio of peak intensities at 1047 cm^−1^ (ordered structure) and 1022 cm^−1^ (amorphous structure) reflects the degree of starch order. The addition of barley pulp significantly reduced this ratio (*p* < 0.05): as the gelatinization degree increased, the 1047/1022 cm^−1^ ratio decreased. Mechanistically, during cooking, the barley pulp promotes water absorption and swelling of starch granules, disrupting intermolecular hydrogen bonds. This loosens the well-ordered starch chains and induces a transition towards an amorphous state, thereby reducing short-range order.

During refrigeration, the short-range order of all samples increased. By day 9, the short-range order of the control group had increased by 8.96%, while increases in the barley pulp treatment groups ranged from 3.36% to 5.56%. This is consistent with the findings of He et al. [23]: refrigeration promotes the association of starch molecular chains and the stacking of helical structures, enhancing orderliness. However, barley pulp significantly inhibited this process. For instance, the highest gelatinization group (BJ90) exhibited only a 3.36% increase after refrigeration, indicating that it delays staling by disrupting the hydrogen bond network and hindering molecular chain rearrangement. Specifically, the gelatinized starch and dietary fiber in the barley pulp may encapsulate starch granules, restricting moisture migration and molecular chain movement. Moreover, a higher degree of gelatinization leads to a greater disruption of the original ordered structure (e.g., the BJ90 group, which had the lowest initial ratio, showed the strongest inhibitory effect). These results are consistent with the thermomechanical property analysis of the dough, confirming that barley pulp inhibits bun staling by regulating intermolecular forces in starch in a graded manner.

### 3.4. Changes in Protein Secondary Structure

The protein secondary structure, analyzed via the FTIR amide I band (1600–1700 cm^−1^), is closely related to gluten network functionality and ultimately influences the texture and storage stability of steamed buns [24,25]. As shown in Table 1, β-sheets and α-helices were the dominant structures (accounting for >60% of the total), followed by random coils, with β-turns being the least abundant. Compared with the control group, the addition of germinated highland barley pulp significantly decreased the initial content of β-sheets and α-helices (*p* < 0.05), while increasing random coils and β-turns. This indicates that barley pulp components (dietary fiber and polyphenols) interfere with gluten network formation, likely through hydrogen bonding and hydrophobic interactions, leading to a more flexible and less ordered protein structure.

During refrigerated storage, the content of both β-sheet and α-helix structures decreased in all samples, indicating progressive gluten degradation. Notably, the barley pulp groups exhibited significantly smaller reductions than the control group. For example, after 9 days, the β-sheet content decreased by 7.63% in the control, whereas the decreases in BJ10, BJ30, BJ60, and BJ90 were 5.88%, 5.52%, 6.22%, 5.27%, and 4.85%, respectively. Similarly, the α-helix content decreased by 15.47% in the control group, compared to only 12.26–14.42% in the barley pulp groups (Table 1). These results demonstrate that barley pulp retards the transformation from ordered (β-sheet, α-helix) to disordered (random coil, β-turn) structures.

The relevance of these changes to bun quality is supported by the texture and specific volume data presented in Section 3.1. A loss of β-sheet and α-helix structures is known to weaken the gluten network, reduce gas retention capacity, and accelerate staling, which manifests as increased hardness and decreased specific volume [26]. In our study, the control group, which had the largest decrease in β-sheet and α-helix contents, also showed the highest increase in hardness (71.76%) and the largest reduction in specific volume (20%) after 9 days. In contrast, the BJ60 group, with moderate retention of ordered secondary structures, exhibited a lower increase in hardness (59.36%) and a smaller reduction in specific volume (18.45%). These observations are consistent with previous reports indicating that preserving β-sheet and α-helix contents helps maintain gluten integrity and delays staling [13].

### 3.5. Changes in in Vitro Digestibility

Data showed that increasing the gelatinization degree of barley pulp significantly decreased RDS (*p* < 0.05) and increased SDS (*p* < 0.05). Furthermore, RS content increased with increasing gelatinization degree: the RS content of the control group was 11.39%, while it increased to 16.52% in the pre-cooking BJ60-1 group (Table 2). The mechanism is related to the breakdown of amylopectin glycosidic bonds during high-temperature pre gelatinization, which generates short amylose chains [27]. Upon cooling, these fragments self-assemble via hydrogen bonds to form enzyme-resistant crystalline structures [28]. Additionally, the RS content in the sauce infiltration layer (maximum 23.23%) was significantly higher than that in the inner crumb (maximum 16.52%) (Table 2). This difference may be attributed to fatty acids in the filling inhibiting starch hydrolysis. Fatty acids can disrupt amylose self-assembly, promoting the formation of ordered crystalline structures that reduce digestibility [29].

**Figure 5:**
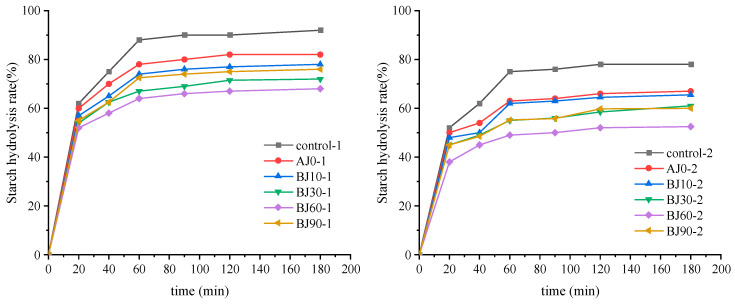
The starch hydrolysis curve of the inner crumb (Sample-1) and sauce infiltration layer (Sample-2) of germinated highland barley pulp barbecued pork bun.

Starch hydrolysis kinetics exhibited a two-phase pattern: a rapid increase from 0 to 60 min, followed by a plateau by 180 min (Figure 5). Notably, the hydrolysis behavior of AJ0 (non-heat-treated barley pulp) differed from that of the BJ groups. In the early phase (0–60 min), AJ0 exhibited a lower hydrolysis rate than the BJ samples because the native starch granules in AJ0 retained an intact crystalline structure, limiting enzyme accessibility. However, between 60 and 180 min, the hydrolysis of AJ0 continued to increase and eventually surpassed that of all BJ groups. This occurred because prolonged incubation caused gradual swelling and disruption of the native granules, whereas the BJ samples—having undergone pre-cooking (gelatinization) followed by refrigerated storage—formed type III resistant starch (retrograded amylopectin) that remained undigested throughout the 180 min assay. Consequently, the final hydrolysis percentage (C∞) was higher for AJ0 than for the BJ samples, consistent with the higher RS content measured in the BJ groups (e.g., BJ60-1 RS = 16.52% vs. AJ0-1 RS = 12.41%, Table 2).

It is important to clarify that the hydrolysis curves in Figure 5 represent the total starch (wheat and barley) in the bun crumb or sauce layer. Since barley pulp solids account for only 8% of the total flour, the majority of the starch is of wheat origin. The observed differences among treatments are therefore primarily driven by how barley pulp components (dietary fiber and pre-gelatinized barley starch) affect the physicochemical environment of wheat starch during steaming and subsequent refrigerated storage. The higher RS content in the BJ groups is attributed to the synergistic effect of pre-cooking (which disrupts barley starch crystallinity) and refrigeration (which promotes the retrogradation of both wheat and barley starch chains), as well as the physical barrier formed by barley dietary fiber [30].

For the BJ90 group, the hydrolysis rate rebounded slightly compared to BJ60, likely due to excessive pre-cooking (91.22% gelatinization), which partially degraded the β-glucan structure, reduced its gel-forming capacity, and caused excessive softening of the fiber network [31]. This is consistent with the lower RS content in BJ90-1 (15.20%) compared to BJ60-1 (16.52%).

### 3.6. Changes in Glycemic Index (GI)

The first-order kinetic constant k is positively correlated with the starch hydrolysis rate; a higher k value indicates a shorter time required to reach the equilibrium hydrolysis percentage C∞. However, there was no significant correlation between the change in k and C∞, possibly due to the heterogeneity in the physicochemical structure of starch granules [32]. As shown in Table 3, the Hydrolysis Index (HI) of the AJ0-1 group with added barley pulp decreased significantly by 32% (*p* < 0.05) compared to the control, consistent with the increase in RS content and α-amylase inhibition results. Pre-gelatinization further reduced HI. The HI for the BJ60-1 group was 60.52, corresponding to a GI value of 60.37, which falls between that of ordinary wheat flour (GI 70–80) and highland barley (GI 25–40), classifying it as a medium GI food (60–69). The medium GI characteristic of highland barley barbecued pork buns is mainly attributed to the gel barrier formed by β-glucan after pre-gelatinization, which inhibits enzyme activity, and the dual regulation effects of dietary fiber: soluble fiber delays hydrolysis by encapsulating starch particles in a hydrophilic gel; (Table 3). Dietary fiber can also adsorb glucose and delay gastric emptying, flattening the postprandial blood glucose response curve [33,34]. The GI value of the sauce infiltration layer (e.g., 53.22 for the BJ60-2 group) was significantly lower than that of the crumb (e.g., 60.37 for the BJ60-1 group). One plausible explanation, based on published in vivo studies, is that lipids from the meat filling may form mixed micelles that delay glucose absorption, while amino acids generated from animal protein digestion may stimulate incretin secretion, thereby reducing postprandial glycemic excursions [35]. However, it should be noted that these mechanisms are derived primarily from in vivo observations and remain speculative for our in vitro system. Therefore, the interpretations above require validation through well-designed animal or human trials. In summary, through the triple mechanisms of β-glucan inhibition, dietary fiber regulation, and lipid-protein interaction, highland barley barbecued pork buns achieve a medium-low GI while maintaining their processing characteristics, demonstrating potential for metabolic health regulation.

### 3.7. Changes in α-Amylase Inhibition

**Figure 6:**
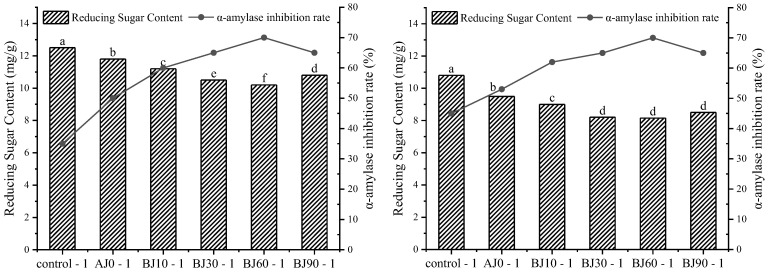
Inhibitory effect of the inner crumb (Sample-1) and sauce infiltration layer (Sample-2) of germinated highland barley pulp barbecued pork bun on α-amylase.

α-Amylase is a key enzyme responsible for breaking down starch into glucose. Inhibiting its activity can delay the hydrolysis of α-1,4-glycosidic bonds and reduce postprandial blood glucose peaks [36]. Adding pre-gelatinized barley pulp significantly increased the α-amylase inhibition rate (*p* < 0.05), and the inhibition rate increased with increasing gelatinization degree: the inhibition rates for the crumb and sauce infiltration layer of the BJ60 group reached 67.72% and 68.80%, respectively (Figure 6). Mechanistically, pre-gelatinization enables the insoluble dietary fiber in highland barley to form a network that encapsulates starch granules, reducing enzyme-substrate contact. However, excessive gelatinization (e.g., BJ90 group) led to a decrease in inhibition rate, likely due to high temperature damage to the starch structure. The inhibition rate of the sauce infiltration layer was generally higher than that of the crumb, possibly because fats in the filling encapsulate starch granules, or because Maillard reaction products hinder enzyme action [37]. These results are consistent with the changes in starch digestibility and GI values, confirming that barley pulp regulates the metabolic characteristics of barbecued pork buns by inhibiting enzyme activity.

### 3.8. Correlation Analysis Between Starch Digestibility and Structural Changes

**Figure 7:**
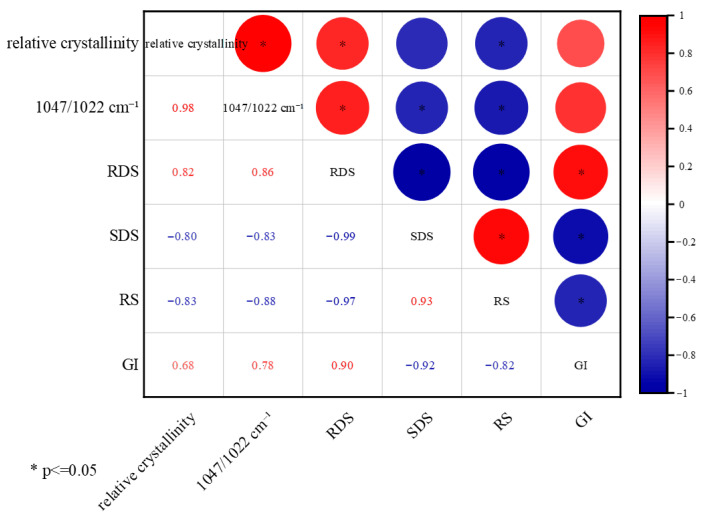
Heatmap for the correlation analysis of starch digestibility and starch structure within the filling of barbecued pork bun, * indicate significant differences (*p* < 0.05).

To explore potential relationships between starch digestibility and structural changes in the bun crumb, correlation analyses were performed among RDS, SDS, RS, GI, relative crystallinity (XRD), and short-range order (1047/1022 cm^−1^) (Figure 7). In this complex food system, which contains both wheat and barley starch as well as dietary fiber from germinated highland barley pulp, no simple linear relationship was observed between starch relative crystallinity and RS content. Similarly, the relationship between short-range order and digestibility fractions was not straightforward. These findings are consistent with the literature, in which both positive and negative correlations between crystallinity and resistant starch have been reported, depending on the starch source, processing conditions, and the presence of other components [38]. The absence of a simple correlation in our study may be attributed to several factors: (i) the crystallinity measured by XRD primarily reflects native A-type crystals of wheat starch, whereas the RS formed during refrigerated storage is predominantly type III (retrograded amylopectin); (ii) the addition of barley dietary fiber may alter crystal morphology and interfere with starch chain reassociation; and (iii) the low proportion of barley pulp (8% solids) means that structural signals are dominated by wheat starch, while digestibility measurements integrate contributions from both starches. Therefore, while the correlation data provide some insights, they should be interpreted with caution. Future studies using isolated starch systems or labeled starches may help decouple these effects.

## 4. Conclusions

The pre-cooking degree of highland barley pulp significantly influenced the quality and digestibility of refrigerated barbecued pork buns. After 9 days, the addition of barley pulp notably delayed quality deterioration, reducing the rate of hardness increase by up to 24.2% compared to the control. Structurally, it decreased starch short-range order (lowered 1047/1022 cm^−1^ ratio, *p* < 0.05) and mitigated the loss of β-sheet and α-helix content. With increasing gelatinization degree (up to 91.22%), digestibility characteristics changed significantly: RDS decreased and RS increased (*p* < 0.05), with RS in the sauce infiltration layer reaching 23.23%. Hydrolysis rates decreased, and the sauce infiltration layer (GI 53.22) digested slower than the crumb (GI 60.37). The BJ60 group exhibited the highest α-amylase inhibition (67.72–68.80%). The relationships between starch structural parameters and digestibility fractions were complex and not simply linear, varying with the degree of pre-cooking and matrix composition. Future studies should include extended shelf-life evaluation (beyond 9 days) under real storage and distribution conditions, as well as large-scale consumer acceptability testing (*n* > 100) to confirm the commercial viability of the optimized formulation. Clinical studies are also needed to validate the in vitro GI predictions and assess long-term metabolic effects.

## Figures and Tables

**Table 1:** Relative contents of protein secondary structure in the inner crumb of germinated highland barley pulp barbecued pork bun during storage period.

Sample	β-Sheet (%)	Random Coil (%)	α-Helix (%)	β-Turn (%)
0d-control	48.94 ± 0.37 ^a^	18.54 ± 0.03 ^c^	25.17 ± 0.05 ^a^	8.05 ± 0.34 ^e^
0d-AJ0	41.19 ± 0.20 ^e^	22.87 ± 0.09 ^a^	21.49 ± 0.11 ^e^	14.45 ± 0.28 ^a^
0d-BJ10	43.27 ± 0.67 ^d^	22.05 ± 0.18 ^a^	23.07 ± 0.08 ^c^	11.61 ± 0.13 ^c^
0d-BJ30	44.33 ± 0.19 ^c^	20.43 ± 0.29 ^b^	23.81 ± 0.02 ^c^	11.43 ± 0.16 ^c^
0d-BJ60	46.09 ± 0.28 ^b^	19.98 ± 0.17 ^b^	24.66 ± 0.07 ^b^	9.27 ± 0.62 ^d^
0d-BJ90	44.85 ± 0.08 ^c^	20.11 ± 0.09 ^b^	22.67 ± 0.13 ^d^	12.37 ± 0.09 ^b^
9d-control	46.17 ± 1.07 ^a^	20.06 ± 0.05 ^e^	21.78 ± 0.06 ^a^	11.19 ± 0.07 ^e^
9d-AJ0	39.18 ± 0.28 ^e^	24.65 ± 0.19 ^a^	18.39 ± 0.03 ^d^	17.78 ± 0.23 ^a^
9d-BJ10	40.88 ± 0.32 ^d^	22.76 ± 0.27 ^b^	19.64 ± 0.02 ^c^	16.72 ± 0.31 ^b^
9d-BJ30	42.46 ± 0.48 ^c^	21.33 ± 0.35 ^c^	20.65 ± 0.03 ^b^	15.56 ± 0.45 ^c^
9d-BJ60	43.67 ± 0.29 ^b^	20.7 ± 0.11 ^d^	20.99 ± 0.12 ^b^	14.64 ± 0.13 ^d^
9d-BJ90	42.09 ± 0.17 ^c^	21.45 ± 0.31 ^c^	19.89 ± 0.05 ^c^	16.57 ± 0.18 ^b^

Different superscript letters indicate significant differences (*p* < 0.05).

**Table 2:** Content of RDS, SDS and RS in the inner crumb (Sample-1) and sauce infiltration layer (Sample-2) of germinated highland barley pulp barbecued pork bun.

Sample	RDS (%)	SDS (%)	RS (%)
Control-1	66.42 ± 0.92 ^a^	22.19 ± 0.46 ^d^	11.39 ± 0.02 ^e^
AJ0-1	61.34 ± 0.89 ^b^	26.25 ± 0.12 ^c^	12.41 ± 0.24 ^d^
BJ10-1	57.87 ± 0.66 ^c^	27.93 ± 0.04 ^b^	14.20 ± 0.17 ^c^
BJ30-1	53.93 ± 0.28 ^d^	31.56 ± 0.78 ^a^	14.51 ± 0.08 ^c^
BJ60-1	51.38 ± 0.13 ^e^	32.10 ± 0.27 ^a^	16.52 ± 0.05 ^a^
BJ90-1	54.81 ± 0.11 ^d^	29.99 ± 0.83 ^a^	15.20 ± 0.12 ^b^
Control-2	55.23 ± 0.42 ^a^	29.40 ± 0.05 ^e^	15.37 ± 0.04 ^d^
AJ0-2	51.39 ± 0.25 ^b^	31.27 ± 0.02 ^d^	17.34 ± 0.21 ^c^
BJ10-2	47.17 ± 0.71 ^c^	32.94 ± 0.09 ^b^	19.89 ± 0.13 ^b^
BJ30-2	43.98 ± 0.68 ^d^	33.43 ± 0.57 ^b^	22.59 ± 0.22 ^a^
BJ60-2	41.76 ± 0.19 ^e^	35.01 ± 0.26 ^a^	23.23 ± 0.19 ^a^
BJ90-2	44.89 ± 0.37 ^d^	32.29 ± 0.18 ^c^	22.82 ± 0.37 ^a^

Different superscript letters indicate significant differences (*p* < 0.05).

**Table 3:** Values of C∞, k, HI and GI for the inner crumb (Sample-1) and sauce infiltration layer (Sample-2) of germinated highland barley pulp barbecued pork bun after 180 min of hydrolysis.

Sample	C∞	k	HI	GI
Control-1	82.92 ± 1.37 ^a^	0.06	100 ^a^	94.40 ^a^
AJ0-1	75.21 ± 0.51 ^b^	0.04	68.48 ± 0.39 ^b^	68.64 ± 0.21 ^b^
BJ10-1	73.03 ± 0.26 ^c^	0.05	65.77 ± 0.31 ^c^	65.16 ± 0.28 ^c^
BJ30-1	66.28 ± 1.07 ^de^	0.05	62.16 ± 0.14 ^e^	61.78 ± 0.12 ^e^
BJ60-1	64.11 ± 1.42 ^e^	0.02	60.52 ± 0.27 ^f^	60.37 ± 0.29 ^f^
BJ90-1	68.74 ± 1.30 ^d^	0.04	63.91 ± 0.03 ^d^	63.78 ± 0.14 ^d^
Control-2	81.45 ± 1.32 ^a^	0.05	76.53 ± 0.11 ^a^	76.26 ± 0.14 ^a^
AJ0-2	72.57 ± 0.19 ^b^	0.04	58.77 ± 0.05 ^b^	58.03 ± 0.07 ^b^
BJ10-2	70.39 ± 0.34 ^c^	0.05	56.34 ± 0.18 ^c^	55.94 ± 0.21 ^c^
BJ30-2	65.44 ± 1.13 ^d^	0.04	55.08 ± 0.39 ^d^	54.71 ± 0.03 ^d^
BJ60-2	62.51 ± 1.02 ^e^	0.03	53.19 ± 0.21 ^e^	53.22 ± 0.22 ^e^
BJ90-2	66.83 ± 1.26 ^d^	0.05	55.72 ± 0.15 ^d^	55.54 ± 0.19 ^c^

Different superscript letters indicate significant differences (*p* < 0.05).

## Data Availability

The original contributions presented in the study are included in the article, further inquiries can be directed to the corresponding authors.
